# Patterns in NIA Health Disparities Research Framework Domains and AD‐Plasma Biomarkers

**DOI:** 10.1002/alz.095372

**Published:** 2025-01-09

**Authors:** Maria Vassilaki, Jeremiah A Aakre, Anna M Castillo, Timothy G. Lesnick, Walter K. Kremers, Alicia Algeciras‐Schimnich, Jonathan Graff‐Radford, David S. Knopman, Thomas H. Mosley, B Gwen Windham, Michael E. Griswold, Val J. Lowe, Clifford R. Jack, Ronald C. Petersen, Prashanthi Vemuri

**Affiliations:** ^1^ Mayo Clinic, Rochester, MN USA; ^2^ Mayo Clinic, Quantitative Health Sciences, Rochester, MN USA; ^3^ Department of Neurology, Mayo Clinic, Rochester, MN USA; ^4^ University of Mississippi Medical Center, Jackson, MS USA; ^5^ Department of Medicine/Geriatrics, The Memory Impairment and Neurodegenerative Dementia (MIND) Center, University of Mississippi Medical Center, Jackson, MS USA; ^6^ University of Mississippi Medical Center, The MIND Center, Jackson, MS USA; ^7^ Department of Radiology, Mayo Clinic, Rochester, MN USA

## Abstract

**Background:**

The NIA Health Disparities Research (NIA‐HD) Framework organizes factors in four domains (i.e., environmental, sociocultural, behavioral, and biological), which work together over the lifetime to influence health and health disparities.

**Method:**

In previous work, we employed a 2‐stage approach using factor and principal component (PC) analyses to identify patterns of factors in the domains of the NIA‐HD Framework. We found three PCs that identified related groupings from more than one NIA‐HD domain. PC1: retirement status, residence type, multivitamin use, heart disease, Charlson comorbidity index, walking/balance difficulties, and Beck Depression Inventory (BDI)‐II questions – mainly from the somatic dimension; PC2: physical activity/exercise, social and group activities, education, occupation, cardiometabolic risk factors, and self‐rated health; PC3: national and state area deprivation index ranking and BDI‐II questions – mainly in the cognitive dimension. Age and sex‐adjusted linear regression models were used to assess the cross‐sectional association of each PC with AD‐plasma biomarkers measured using the Quanterix Simoa platform in the Mayo Clinic Study of Aging (MCSA) participants. AD‐plasma biomarkers included Aβ 42/40, phosphorylated‐tau 181 (p‐tau181), plasma glial fibrillary acidic protein (GFAP), and neurofilament light chain (NfL). All analyses were considered statistically significant at a p <0.05.

**Result:**

There were 1300 cognitively unimpaired participants, 50 years old or older, with available plasma biomarkers (mean age (SD) 68.6 (9.5) years; 49.1% were female) at the current study baseline. PC1 and PC3 were associated with higher NfL and p‐tau 181 values (p<0.05). PC1 and PC2 were associated with higher GFAP values (p<0.05). No PCs were associated with the Aβ 42/40 ratio. Findings were mostly similar after additionally adjusting for global cognitive performance and chronic kidney disease.

**Conclusion:**

We found that patterns in a wide range of environmental, sociocultural, behavioral, and biological factors were associated with AD plasma biomarkers. Longitudinal studies are warranted to validate findings and support multi‐faceted approaches to address health disparities and achieve disease delay and prevention.

